# Supplementary material for: Linking plasmid-based beta-lactamases to their bacterial hosts using single-cell fusion PCR
Source: eLife. 2021 Jul 20;10:e66834. doi: 10.7554/eLife.66834 (PMC8294855; doi:10.7554/eLife.66834)
Supplement: Transparent reporting form [file elife-66834-transrepform.docx]

***eLife’s* transparent reporting form**

We encourage authors to provide detailed information *within their submission* to facilitate the interpretation and replication of experiments. Authors can upload supporting documentation to indicate the use of appropriate reporting guidelines for health-related research (see [EQUATOR Network](http://www.equator-network.org/%20)), life science research (see the [BioSharing Information Resource](https://biosharing.org/)), or the [ARRIVE guidelines](http://www.plosbiology.org/article/info:doi/10.1371/journal.pbio.1000412) for reporting work involving animal research. Where applicable, authors should refer to any relevant reporting standards documents in this form.

If you have any questions, please consult our Journal Policies and/or contact us: [editorial@elifesciences.org](mailto:editorial@elifesciences.org).

**Sample-size estimation**

- You should state whether an appropriate sample size was computed when the study was being designed
- You should state the statistical method of sample size computation and any required assumptions
- If no explicit power analysis was used, you should describe how you decided what sample (replicate) size (number) to use

Please outline where this information can be found within the submission (e.g., sections or figure legends), or explain why this information doesn’t apply to your submission:

In our manuscript, we were focused on developing a method and providing a proof of concept, as opposed to proving a hypothesis, and thus we did not perform a power analysis to decide the appropriate sample size for our study. We experimentally validated that OIL-PCR in three distinct biological replicates to show that it is sensitive to detect as few as 20 cells and accurate with up to 99% of reads assigning correct associations. In our analysis we used a cut-off threshold of 0.5% for reporting positive associations, performing the experiment both singleplexed and multiplexed, as well as targeting 7 genes to confirm our positive result. Additionally we plot the raw data in figure 1b, 2c, 3b, and fig 3- Supp 1 for critical analysis of our results.

**Replicates**

- You should report how often each experiment was performed
- You should include a definition of biological versus technical replication
- The data obtained should be provided and sufficient information should be provided to indicate the number of independent biological and/or technical replicates
- If you encountered any outliers, you should describe how these were handled
- Criteria for exclusion/inclusion of data should be clearly stated
- High-throughput sequence data should be uploaded before submission, with a private link for reviewers provided (these are available from both GEO and ArrayExpress)

Please outline where this information can be found within the submission (e.g., sections or figure legends), or explain why this information doesn’t apply to your submission:

We include information of biological and technical replicates throughout the manuscript for each experiment. We use stool samples from three donors across two species as biological replicates and perform each experiment with at least three technical replicates. We repeat experiment. Outliers are plotted and discussed within the manuscript, as well as the cut-off we used of 0.5% of total reads in assigning a possible detection. Sequencing data has been uploaded to the SRA, accession number PRJNA701446

**Statistical reporting**

- Statistical analysis methods should be described and justified
- Raw data should be presented in figures whenever informative to do so (typically when N per group is less than 10)
- For each experiment, you should identify the statistical tests used, exact values of N, definitions of center, methods of multiple test correction, and dispersion and precision measures (e.g., mean, median, SD, SEM, confidence intervals; and, for the major substantive results, a measure of effect size (e.g., Pearson's r, Cohen's d)
- Report exact p-values wherever possible alongside the summary statistics and 95% confidence intervals. These should be reported for all key questions and not only when the p-value is less than 0.05.

Please outline where this information can be found within the submission (e.g., sections or figure legends), or explain why this information doesn’t apply to your submission:

We performed statistical tests when comparing rarefaction curves in the lysozyme treatments (Mann-Whitney with FDR correction). P-values are reported in the figures, and the number of replicates is disclosed both in the text and the figure legends.

(For large datasets, or papers with a very large number of statistical tests, you may upload a single table file with tests, Ns, etc., with reference to sections in the manuscript.)

**Group allocation**

- Indicate how samples were allocated into experimental groups (in the case of clinical studies, please specify allocation to treatment method); if randomization was used, please also state if restricted randomization was applied
- Indicate if masking was used during group allocation, data collection and/or data analysis

Please outline where this information can be found within the submission (e.g., sections or figure legends), or explain why this information doesn’t apply to your submission:

We did not use group allocation in this study

**Additional data files (“source data”)**

- We encourage you to upload relevant additional data files, such as numerical data that are represented as a graph in a figure, or as a summary table
- Where provided, these should be in the most useful format, and they can be uploaded as “Source data” files linked to a main figure or table
- Include model definition files including the full list of parameters used
- Include code used for data analysis (e.g., R, MatLab)
- Avoid stating that data files are “available upon request”

Please indicate the figures or tables for which source data files have been provided:

Source data was provided on the github repository for fig 1b, fig 1c, fig 2c, and Fig 1—Supp 6. https://github.com/pjdiebold/OIL-PCR_Linking_plasmid-based_beta-lactamases
